# HIV-1 Env Heterogeneity: Cleavage, Trafficking, and Antigenic Consequences for Virions and Infected Cells

**DOI:** 10.3390/v18080811

**Published:** 2026-07-24

**Authors:** Dania M. Figueroa Acosta, Sara Khaleeq, Svenja Weiss, Tony R. Valencia, Guy Mason, Benjamin K. Chen

**Affiliations:** Division of Infectious Diseases, Department of Medicine, Icahn School of Medicine at Mount Sinai, New York, NY 10029, USA

**Keywords:** HIV, envelope glycoprotein, endocytic recycling, intracellular trafficking, therapeutics, cytoplasmic tail

## Abstract

The HIV-1 Env glycoprotein mediates both cell-free and cell-to-cell viral transmission and represents the primary target for protective humoral immune responses. Studies examining antibody neutralization of cell-free and cell-to-cell HIV transmission have found that cell-to-cell transmission is more resistant to neutralization. This resistance may be explained in part by antigenically distinct Env populations on virions and infected cells. Cell-surface Env may be more heterogeneous due to variations in cleavage, glycosylation, and conformational state. Nevertheless, the mechanisms that maintain antigenically distinct Env populations at the cell surface and on virions remain unclear, despite virion assembly occurring at the plasma membrane. In this focused review, we consider how Env endocytosis and recycling influence Env incorporation into virions and antibody recognition. We further consider how Env cleavage may influence trafficking and endocytic fate. Given the central role of Env’s cytoplasmic tail in engaging endosomal trafficking pathways, we review emerging structural models of the CT and discuss how its organization, symmetry, and conformational flexibility may contribute to Env trafficking and intracellular sorting. We also discuss how heterogeneous Env populations may influence antibody susceptibility. Finally, we review therapeutic strategies, including combinatorial antibodies and small-molecule Env modulators, that may enhance antibody recognition of infected cells and virions.

## 1. Introduction

The human immunodeficiency virus (HIV) envelope glycoprotein (Env) mediates entry into CD4-expressing immune cells and drives both cell-free and cell-to-cell modes of viral transmission [1,2,3]. As the sole viral protein exposed on the surface of infected cells and virions, Env is the primary target of antibody-mediated immune responses. Antibodies can inhibit HIV infection by blocking receptor engagement, preventing co-receptor binding (CCR5/CXCR4), or interfering with membrane fusion [4,5,6]. Env-specific antibodies can further trigger Fc-dependent effector functions that clear infected cells [5,6,7,8]. Additionally, neutralizing antibodies can block cell-to-cell transmission through virological synapses, where cell-surface Env is the key target [9,10]. However, cell-to-cell transmission is generally more resistant to antibody-mediated inhibition than cell-free infection due [11,12,13,14], at least in part, to differences in Env presentation, conformation, and accessibility that influence antibody recognition.

**Figure 1:**
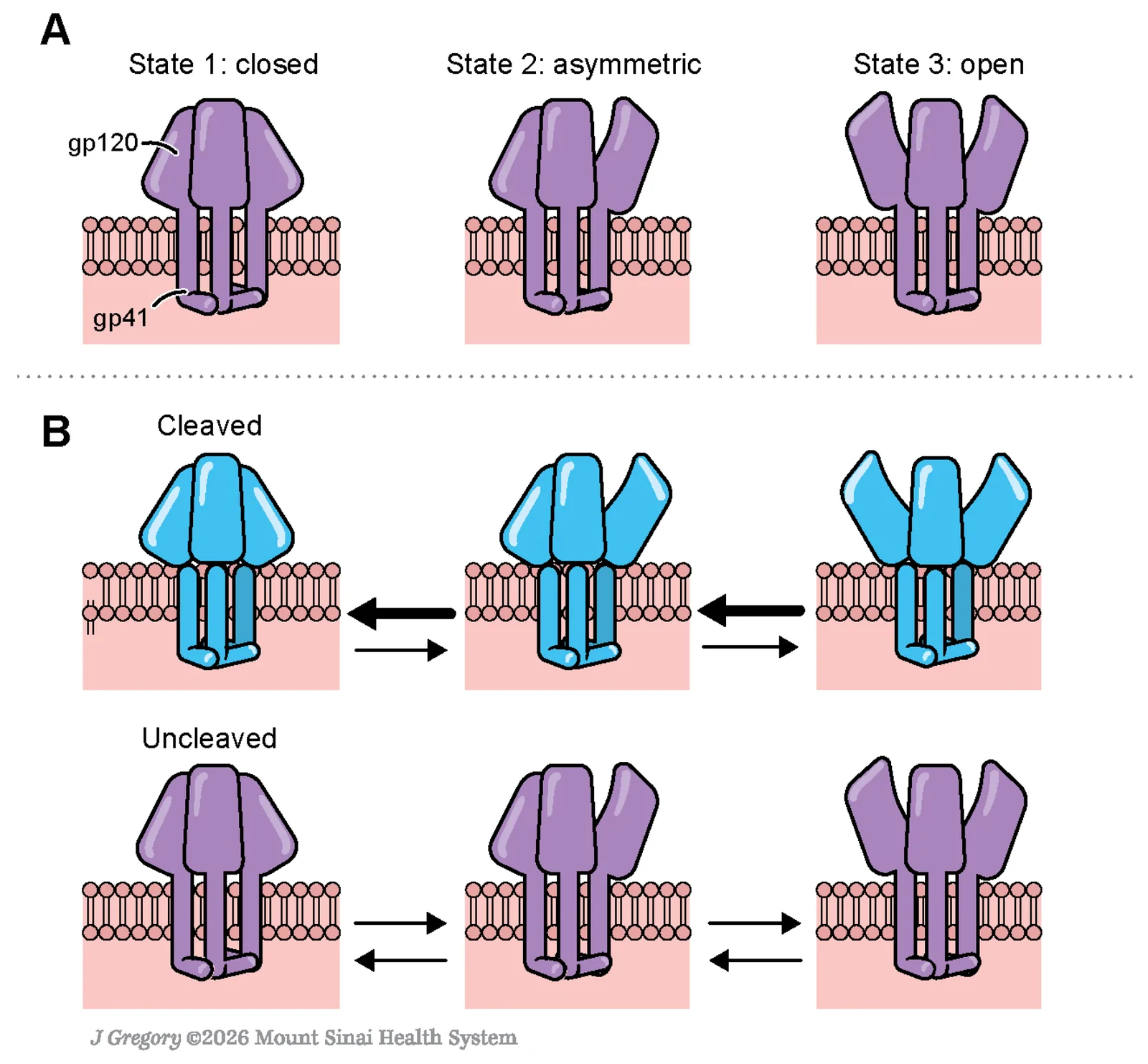
Conformational states and cleavage-dependent dynamics of HIV-1 Env. (**A**) Schematic representation of the conformational states sampled by membrane-bound HIV-1 Env (only one Env form is shown for simplicity) [21,22,23,24]. (**B**) The effects of proteolytic cleavage on the conformational dynamics of cleaved Env (blue, top) and uncleaved Env (purple, bottom) are illustrated. Dark and light arrows indicate predominant and alternative conformational states sampled by each Env form, respectively [27,28,29,30,31]. Illustration by Jill Gregory. Used with permission of ©Mount Sinai Health System.

HIV Env has evolved to be adept at immune evasion by generating high sequence diversity [15,16] and maintaining a dense glycan shield [17,18,19,20]. Additionally, Env can spontaneously transition between three conformational states: state 1, a prefusion closed trimer; state 2, an intermediate asymmetric state in which one or two protomers are closed; and state 3, the pre-hairpin intermediate, in which all protomers are closed (Figure 1A) [21,22,23,24]. Another source of Env structural diversity arises from post-translational processing during biosynthesis and the differential display of antigenically distinct Env populations on virions and infected-cell surfaces. Env is synthesized as a precursor glycoprotein, gp160, and subsequently cleaved into the noncovalently associated subunits gp120 and gp41 to form the fusion-competent trimer [25,26,27]. Cleavage is required to generate the fusion-competent trimer and stabilizes a more compact prefusion conformation with an antigenic profile distinct from that of uncleaved Env (Figure 1B) [27,28,29,30,31]. While virions are preferentially enriched for cleaved, fusion-competent trimers, infected-cell surfaces display a heterogeneous mixture of both cleaved and uncleaved Env, together with multiple conformational states [32,33,34,35]. This differential distribution is of particular interest because it may influence antibody recognition, immune evasion, and the relative susceptibility of cell-free and cell-to-cell viral transmission to neutralization. The mechanisms that establish and maintain these distinct Env populations, however, remain poorly understood.

This review examines how intracellular trafficking, proteolytic processing, and intrinsic conformational dynamics cooperate to generate antigenically distinct Env populations on infected cells and virions. We discuss emerging evidence that structural features of the gp41 cytoplasmic tail influence Env trafficking and sorting and consider how heterogeneous Env populations affect antibody recognition and viral transmission. Lastly, we review some therapeutic strategies aimed at modulating Env antigenicity to enhance immune-mediated clearance of HIV-infected reservoir cells.

## 2. Post-Endocytic Trafficking and Differential Sorting of Distinct HIV Env Populations

The heterogeneous population of Env on the surface of infected cells reflects, in part, incomplete proteolytic processing during Env synthesis. Cleavage of gp160 into gp120-gp41 not only primes Env for fusion [25,26,27], but also stabilizes a more compact, prefusion conformation with a distinct antigenic profile (Figure 1B) [27,28,29,30,31]. Consequently, inefficient cleavage in the Golgi results in the co-expression of structurally and antigenically divergent cleaved and uncleaved trimers at the cell surface [32,33,34,35]. However, the distribution and persistence of these Env populations are not solely determined by biosynthesis, as Env is rapidly internalized following surface expression and trafficked through endocytic recycling pathways [36,37,38,39,40,41,42,43,44,45]. This process is mediated in part by a conserved YXXL AP-2 binding motif within Env’s cytoplasmic tail (CT) [45,46,47]. Additional motifs within the CT have also been implicated in regulating Env trafficking and recycling pathways [39,43,48,49,50,51,52].

**Figure 2:**
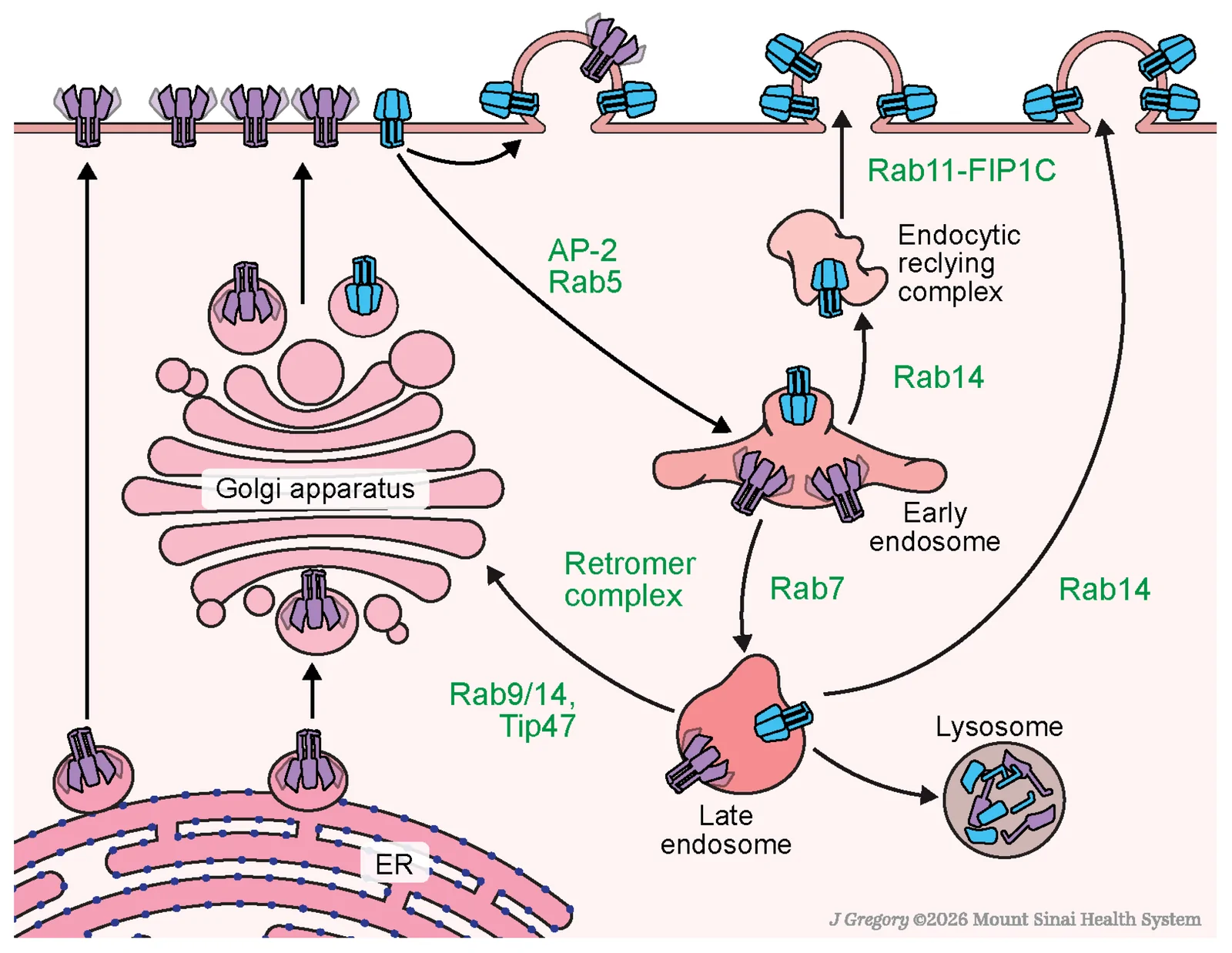
Intracellular trafficking and recycling pathways of HIV-1 envelope glycoprotein (Env). Schematic representation of the intracellular trafficking and recycling pathways of HIV-1 Env between the plasma membrane and endosomal compartments. Newly synthesized Env gp160 (purple) is transported from the endoplasmic reticulum (ER) through the Golgi apparatus to the plasma membrane [25,55,56]. During transport through the trans-Golgi network (TGN), gp160 is cleaved by furin-like proteases into mature gp120–gp41 trimers (blue) [25,26]. Following surface expression, Env may become incorporated into budding virions or undergo clathrin-mediated endocytosis through interactions with adaptor protein 2 (AP-2) and Rab5, resulting in internalization into early endosomes [38,45,46,47]. From early endosomes, Env may recycle back to the plasma membrane through Rab11-FIP1C and Rab14-associated pathways [39,41], traffic retrogradely to the Golgi through the retromer complex [37], or progress to late endosomes via Rab7 [38,40,42]. From late endosomes, Env may undergo lysosomal degradation, recycle back to the cell surface via a Rab14-associated pathway [40,42,43] or traffic to the TGN via Rab9/14- and Tip47-assocaited pathways [57,58]. Illustration by Jill Gregory. Used with permission of ©Mount Sinai Health System.

Following internalization from the plasma membrane, Env has been reported to engage in several intracellular trafficking pathways (Figure 2), including trafficking through late endosomes, the endocytic recycling complex, and the retrograde transport pathway [52,53]. One pathway directs Env to late endosomal and lysosomal compartments, a process involving endosomal Rab GTPases [38,40,42]. While some studies suggest that this route primarily targets Env for degradation, Hoffman et al. demonstrated that Env can colocalize with late endosomal markers without undergoing degradation [38]. These findings raise the possibility that such compartments may not function exclusively as classical degradative lysosomes but instead may represent components of the T cell secretory pathway that serve as reservoirs for Env trafficking and recycling [40]. Additional support for engagement in secretory pathways is provided by Jolly et al., who found that endocytosed Env engages the regulatory secretory pathway in CD4+ T cells to facilitate polarized Env delivery and cell-to-cell spread through the virological synapse (VS) [54].

A second route involves recycling of internalized Env back to the plasma membrane via the endosomal recycling complex (ERC), which promotes efficient Env incorporation into virus particles (Figure 2) [39,41]. As virion assembly is thought to occur at specialized plasma membrane microdomains [59,60,61], recycling through the ERC may contribute to the selective redistribution of Env populations to focal viral assembly sites. This process may occur both at plasma membrane budding sites and at VSs where Gag is pre-assembled. Consistent with this model, Buttler et al. observed enrichment of Env at the necks of budding virions and attributed this localization to vesicular retention and CT-dependent preferential capture of Env by late-stage Gag lattices [62]. Similarly, rapid recycling of Env, but not Gag, at established VSs, where polarized virion assembly occurs, suggests that endocytic recycling contributes to the maintenance and replenishment of Env at cell-to-cell transmission sites [63]. Importantly, the mechanisms regulating ERC-dependent trafficking appear to exhibit cell-type specificity. The Rab11a family interacting protein 1C (FIP1C), which interacts with Rab14 to mediate CT-dependent recycling of Env from the ERC to the plasma membrane [41,44], was found to be important for H9 cells but had minimal effects on packaging in primary CD4+ T cells, and other laboratory-adapted cell lines [43]. Consistent with a role for ERC-mediated trafficking, the tubular recycling endosome has also been implicated in regulating Env incorporation into virions in a cell-type-dependent manner [64].

A third proposed route involves retrograde trafficking of internalized Env back to the Golgi (Figure 2) [37]. Groppelli et al. demonstrated that RNAi inhibition of retromer, a complex involved in retrograde transport and endosomal sorting, reduced the association of a gp41 CT fusion protein with a fluorescent Golgi marker in HeLa cells [37]. Additionally, disruption of retromer increased Env expression on the surface of infected cells and virions in both HeLa and Jurkat T cells, supporting the possibility that Env can traffic from the plasma membrane back to the Golgi [37]. Additional evidence for retrograde trafficking emerged from studies identifying the YW_802_ motif within the CT as a potential interaction site for TIP47, an adaptor protein involved in late endosome trafficking back to the trans-Golgi network (TGN) [57,58,65]. However, this pathway is not yet well defined, as subsequent studies were unable to replicate these claims by either TIP47 knockout or overexpression [36]. Retrograde trafficking of Env theoretically could return Env to the Golgi, where furin, furin-like proteases, and endoglycosidases reside. Whether the return of Env to this compartment results in additional post-translational processing remains unknown.

Despite the identification of multiple trafficking routes for Env, how these pathways influence the sorting of distinct Env populations has not been carefully examined. Recent work by Figueroa Acosta et al. found that disruption of Env internalization alters the cell-surface composition of Env, disproportionally increasing the amount of uncleaved relative to cleaved Env [35]. These findings suggest that Env cleavage and/or conformational state may influence internalization and/or post-endocytic trafficking, thereby contributing to the differential distribution of cleaved and uncleaved Env at the infected-cell surface. Recent advances in our understanding of the Env CT structure now provide an opportunity to explore how its conformation may contribute to Env’s diverse trafficking pathways.

## 3. Dynamics of the gp41 Cytoplasmic Tail and Implications for Env Trafficking

**Figure 3:**
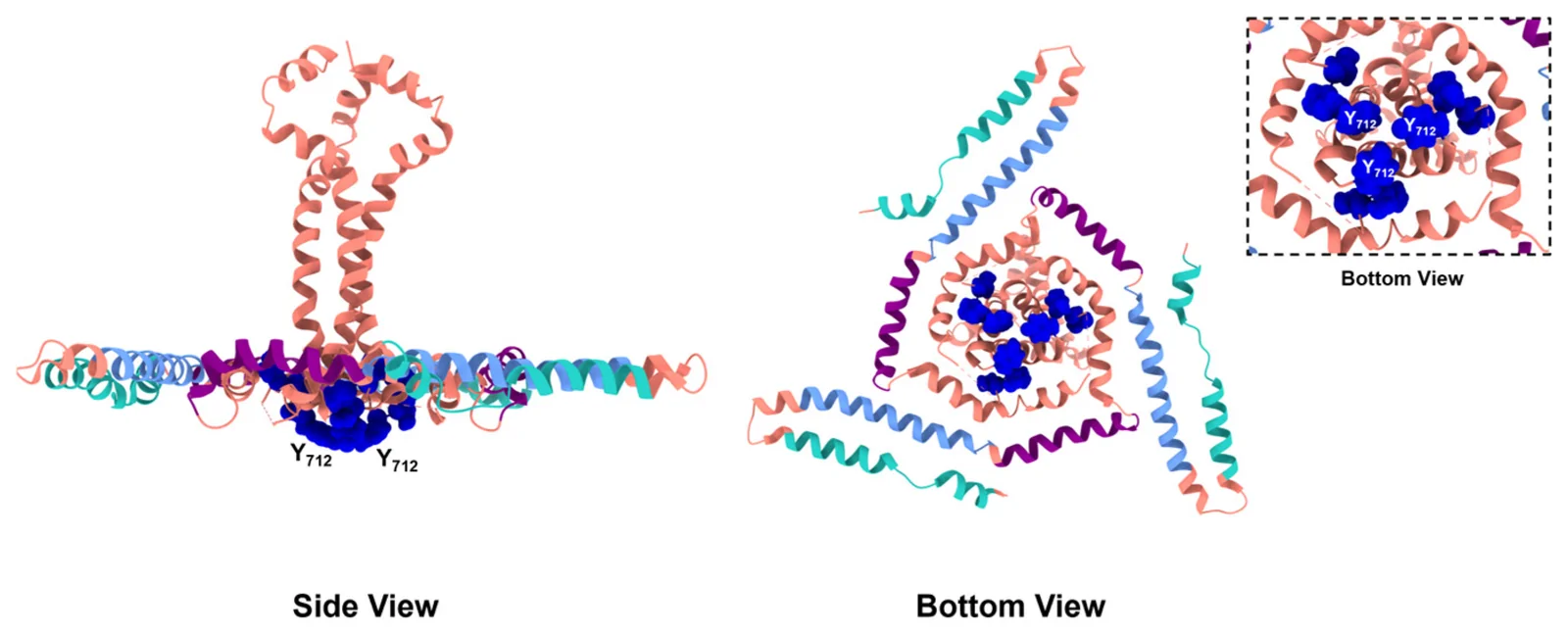
Structural model of HIV-1 Env gp41 membrane-proximal external region (MPER), transmembrane domain, and cytoplasmic tail. Side (**left**) and bottom (**right**) views of the HIV-1 Env gp41 membrane-proximal external region (MPER), transmembrane domain (TMD), and cytoplasmic tail are shown in salmon ribbon representation. The structural model was generated from coordinates of PDB entry 7LOI, which captures the baseplate-like conformation of the gp41 ectodomain–TMD–cytoplasmic tail assembly as a homotrimer [71]. The membrane-proximal YXXL internalization motif (residues 712–715, HXB2 numbering) [45,46,47] is highlighted in blue. The inset shows an enlarged image of the YXXL motif when viewed along the 3-fold axis, and Y712 residues in each protomer are labeled.

Given that multiple endocytic and recycling motifs reside within the CT, understanding its structural organization is critical for determining how Env engages diverse intracellular trafficking pathways. However, the structure of Env’s CT remains poorly defined, largely due to its membrane-embedded nature. Most insights derive from nuclear magnetic resonance (NMR) analyses, mutational studies, and computational modeling [66,67,68,69]. A prevailing model proposes that the CT forms a structured baseplate along the cytoplasmic leaflet of the plasma membrane, contributing to the stabilization of the transmembrane domain (TMD) [70,71] (Figure 3). Central to this proposed organization is the arrangement of the lentiviral lytic peptides (LLPs), amphipathic helices, positioned at the membrane interface. Earlier models suggested that these helices either adopted a transmembrane conformation [72,73] or lay parallel to the cytoplasmic leaflet [66]. In contrast, more recent work by Piai and colleagues proposes that the LLP-derived helical hairpins form inner and outer rings that constrain scissor-like motion of the TMD [70], supporting a model of the CT that is more structurally dynamic.

The dynamic properties of the TMD further support a model in which structural flexibility extends into the CT. Hollingsworth IV et al. demonstrated that residues near the TMD-CT junction can adopt kinked conformations that, in molecular dynamic simulations, permit transient hydration of the trimeric transmembrane core by water and ions [74]. These conformational changes were associated with local membrane deformation and increased flexibility of the gp41 TMD, suggesting a potential mechanism for coupling membrane dynamics to Env conformation [74]. Beyond the TMD, Murphy et al. demonstrated that the CT is not uniformly flexible but instead contains regions that differ in their structural organization and membrane association [66]. In agreement, Piai et al. observed that the first 28 residues of the CT, encompassing the Kennedy sequence (KS), display enhanced mobility compared to the remainder of the tail [71]. Together, these studies support a model in which the CT is highly dynamic and context-dependent. Consistent with this structural flexibility, Majumder et al. used computational and AI-based modeling and found that individual protomers within the prefusion-associated MPER-TMD trimer can adopt distinct conformations [75], supporting a model in which the CT is not constrained to a symmetric architecture. These findings further raise the possibility that previously proposed models of the CT may not be mutually exclusive, but instead reflect distinct conformational states stabilized under different structural or membrane environments. The factors governing these transitions remain unclear and may be influenced by changes in Env conformation, local membrane composition, or other contextual features of the viral and cellular membrane landscape. In support of this, Steckbeck et al. found that exposure of the KS is largely inaccessible on virion-associated Env but becomes accessible under certain cellular or experimental conditions [68]. Such observations suggest that structural flexibility within the CT may regulate the accessibility of trafficking determinants embedded within the tail.

Despite this structural flexibility, CT geometry remains functionally important. Groves et al. examined whether the trimeric structure of the Env CT is necessary for lattice trapping of Env and incorporation into virus assembly sites [76]. While the CT was necessary for efficient incorporation into virus-like particles (VLPs), this process was not oligomerization-dependent [76]. However, the CT’s structural geometry remained important as compensatory mutations in the matrix (MA) that restore incorporation of native Env trimers (e.g., MA-34VI rescues MA-12LE) [77]) failed to rescue the incorporation of monomeric Env-CT [76]. Notably, monomeric Env-CT remained partially competent for incorporation, likely through lattice trapping and passive incorporation [76]. Based on these findings, the authors propose that the CT may transition between baseplate-like and linear conformations to mediate lattice interactions [76].

This structural flexibility may also have implications for trafficking. In the baseplate-like conformation, the spatial arrangement of adjacent tyrosine and leucine residues may impose steric constraints that limit direct engagement of the YXXL motif with the μ2-subunit of AP-2, raising the possibility that additional transient conformational rearrangements are required for efficient adaptor engagement (Figure 3). Although direct experimental evidence for such rearrangements in HIV Env is currently lacking, structural studies of EGFR- and TGN38-derived peptides have demonstrated that YXXL motifs adopt an extended conformation when bound to the AP-2 μ2 domain [78]. Consistent with this model, the AP-2 peptide-binding groove is predicted to require presentation of the internalization motif as an accessible, relatively unstructured region, and AP-2 engagement has been reported to increase under denaturing conditions that promote conformational flexibility [78]. Together, these observations support a model in which AP-2 engagement requires transient structural rearrangements that expose the YXXL motif in an extended, accessible conformation permissive for adaptor recruitment.

Collectively, these data indicate that the CT is not merely a structural scaffold for Env incorporation, but a regulatory domain that influences Env organization and trafficking within infected cells. Consistent with this, truncations and mutations of the CT have been shown to alter the conformation of Env’s ectodomain, promoting more open Env conformations [79,80,81]. Together with evidence that CT residue exposure may govern trafficking of internalized Env, these findings support models in which CT structural flexibility may regulate access to trafficking determinants. An outstanding question, however, is whether perturbations to the gp120 subunit can allosterically influence CT structure, thereby altering key residue positioning and modulating Env’s intracellular trafficking.

## 4. Native-like HIV Env: Cleavage, Structural Asymmetry and Functional Heterogeneity

The first structural models of the trimeric Env were pioneered using stabilized, cleaved Env trimers that represent a native conformational state of Env [82,83]. Additionally, many cryo-EM studies examine transmitted/founder (T/F) clones such as BG505 and AMC011 and employ antibody-based purification strategies that enrich for well-cleaved, state 1-like trimers [84,85,86,87]. These Env’s are efficiently recognized by broadly neutralizing antibodies and have provided a strong foundation for the rational design of Env-based immunogens for vaccine development. Among the most widely studied immunogens are SOSIP trimers, a stabilized form of Env that incorporates a disulfide bond between gp120 and gp41 (SOS) and an isoleucine-to-proline substitution (IP) in gp41 that stabilizes the prefusion conformation [88,89]. These constructs have proven highly valuable for structural studies and have been shown to elicit autologous neutralizing antibody responses in animal and clinical studies [90,91,92,93,94]. However, despite substantial trimer optimization, induction of broadly neutralizing antibody responses remains a major challenge for SOSIP-based immunogens. Subsequent studies demonstrated that soluble Env trimers can expose non-neutralizing epitopes, including immunodominant glycan holes, that divert the immune response away from conserved neutralizing determinants [92,95,96]. Consequently, alternative immunogen strategies are being explored, including membrane-bound Env constructs designed to better recapitulate native Env presentation and reduce off-target antibody responses [97,98,99]. In support, Parks et al. reported in a phase 1 clinical trial (Clinical Trials.gov: NCT05217641) that mRNA-encoded membrane-bound Env reduced off-target responses and enhanced autologous neutralizing responses compared to mRNA-encoded soluble Env [99]. Additional efforts have focused on developing germline targeting SOSIP designs capable of engaging broadly neutralizing antibody (bNAb) precursor B-cells [100].

Moreover, these immunogen strategies are predicted on the assumption that cleaved Env most closely represents the native antigenic target for eliciting anti-Env antibodies. Proteolytic cleavage not only stabilizes the prefusion trimer but also shifts the conformational equilibrium toward more closed, state 1-like conformations, thereby altering the relative occupancy of distinct Env states (Figure 1B). Cleavage additionally influences glycan processing by limiting access to Golgi glycoenzymes, resulting in a glycan shield enriched in less processed glycans [101]. In turn, glycans themselves also contribute to the stabilization of the closed Env conformation [19,102]. Zhang et al. recently showed that deletion of a glycan in the first variable loop or alterations to the fusion peptide proximal region stabilize state 1 and reduce spontaneous gp120 shedding [102]. Together, these findings highlight the close interplay between proteolytic cleavage, glycan composition, and Env conformational stability. This complexity may complicate efforts to define a single native-like Env structure and has important implications for immunogen design and therapeutic targeting.

Beyond cleavage- and glycan-dependent effects, accumulating structural evidence suggests that native Env may exhibit greater asymmetry than originally appreciated. T/F HIV clones, previously thought to adopt predominantly closed conformations [103,104], have been shown to display intrinsic asymmetry [86,105]. Parthasarathy et al. analyzed a panel of T/F clones and found that 6 of 13 strains were sensitive to antibodies that recognized epitopes normally associated with more open Env conformations [105]. Moreover, they observed that viruses evolved toward more closed conformations over time, suggesting progressive immune-driven stabilization of state 1-like Env [105]. Further supporting a functional role for asymmetry, Wang et al. demonstrated that imposing symmetry on Env trimers introduced steric clashes between CD4 and the asparagine 301 residue on the protomer with the smallest opening angle [86]. These findings suggest that natural asymmetry may relieve such constraints and facilitate engagement of multiple CD4s during viral entry [86]. Together, these findings indicate that structural asymmetry may serve a functional role during viral entry and challenge the assumption that a single symmetric state fully represents native Env.

This consideration also extends to uncleaved Env, which is often excluded from vaccine design because it is fusion incompetent and only minimally incorporated into virions. However, recent work from Figueroa Acosta et al. demonstrated that uncleaved Env can engage CD4 on the surface of infected cells and promote cell-to-cell transmission [35]. Together with previous studies showing that uncleaved Env can bind CD4 [29,106], these findings suggest that uncleaved Env may contribute to the initiation of cell-to-cell transmission through productive CD4 engagement. The authors further observed that increasing the proportion of uncleaved Env at the cell surface altered bNAb activity in cell-free infection assays, despite continued preferential incorporation of cleaved Env into virions [35]. These observations suggest that alterations in the composition of Env populations on infected cells can influence antibody-mediated inhibition even when virion-associated Env remains predominantly cleaved. Coupled with emerging models of HIV entry in which multiple Env trimers cooperate during fusion to form the entry claw [23], these findings raise the possibility that antibodies bound to uncleaved Env could indirectly impair the function of neighboring cleaved, fusion-competent trimers within the multimeric entry complex, thereby enhancing inhibition of viral entry. Altogether, these findings challenge the assumption that uncleaved Env is biologically irrelevant and suggest that antibody-targeting of uncleaved Env may be beneficial in limiting HIV infection. Collectively, these findings suggest that heterogeneous Env populations are not merely byproducts of inefficient processing but may play functional roles in viral transmission or immune evasion. Consequently, therapeutic strategies that alter Env cleavage, conformational occupancy, or trafficking may influence not only viral entry but also the efficacy of Env-targeted antibodies.

## 5. Consequences of Env Heterogeneity and Therapeutic Strategies

The sparse distribution, antigenic heterogeneity, and intrinsic asymmetry of Env present challenges for antibody-mediated targeting of both virions and infected cells. Individual antibody specificities may preferentially recognize only a subset of Env populations, limiting the breadth and efficiency of antibody recognition. These challenges may be particularly pronounced when targeting infected cells, as the broader spectrum of antigenically distinct Env populations displayed on infected cells, compared with virions, may partly contribute to the increased resistance, and in some settings, incomplete neutralization of cell-to-cell transmission by bNAbs [14,35,107]. Additionally, asymmetry of protomers within a trimer may also affect antibody binding and functional avidity at the cell surface. These challenges may be particularly relevant for Fc-dependent effector functions directed against infected cells, where efficient Fcγ receptor engagement depends on sufficient antibody occupancy and the spatial organization of Fc domains generated upon Env binding [108]. Consequently, strategies that broaden recognition, increase functional avidity, or alter Env presentation are increasingly being explored.

To overcome limitations imposed by Env heterogeneity and low density, several strategies have been proposed. One approach involves combining antibodies targeting distinct Env epitopes and conformational states [109,110,111,112]. Tuyishime et al. evaluated combinations of two to six neutralizing and non-neutralizing antibodies and found that a three-monoclonal antibody (mAb) combination consisting of A32 (targeting constant regions 1 and 2), DH511.2K3 (targeting MPER), and PGT121 (targeting third variable loop) mediated rapid and robust killing of infected cells, including those infected with neutralization-resistant and reservoir-derived viruses [112]. The inclusion of both neutralizing and non-neutralizing antibodies further suggests that therapeutic benefit may extend beyond direct neutralization and instead reflect improved recognition of heterogeneous Env populations coupled with enhanced Fc-mediated effector activity. Collectively, these findings support the concept that simultaneous engagement of multiple Env conformations may overcome epitope masking and broaden antibody activity across diverse Env populations.

Beyond simple combinations, engineered bispecific and multispecific antibody platforms have emerged as approaches to reduce dependence on high local epitope density [113,114,115,116]. By simultaneously targeting multiple Env epitopes, or bridging Env with effector cell receptors, these constructs enhance immune synapse formation and promote more efficient killing of infected cells, even under conditions of limited Env accessibility or conformational heterogeneity. Such approaches may therefore overcome constraints imposed by heterogeneous Env populations by improving both breadth of recognition and functional avidity.

**Figure 4:**
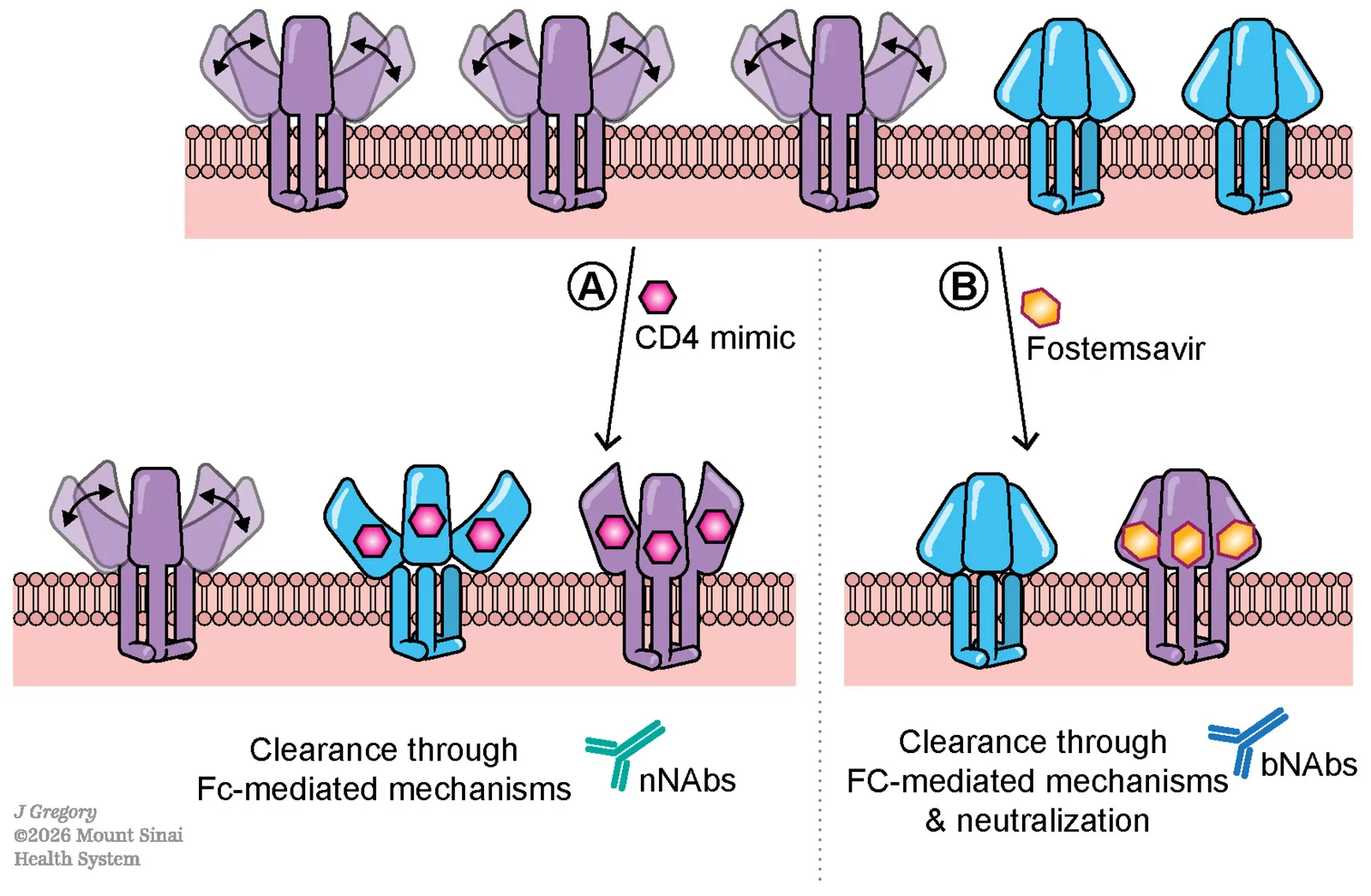
Therapeutic strategies targeting HIV-1 Env to modulate antigenicity and viral transmission. Distinct Env conformations and maturation states are depicted, highlighting their influence on antigenic exposure and immune recognition. (**A**) CD4 mimetics that induce open Env conformations, exposing CD4-induced epitopes and permitting broader recognition of non-neutralizing antibodies (nNAbs), thereby sensitizing infected cells to Fc-mediated clearance [117,118,119,120,121]. (**B**) Small-molecule Env inhibitors, including fostemsavir, stabilize closed conformations and prevent CD4-induced exposure of otherwise hidden epitopes, could potentially synergize with broadly neutralizing antibodies (bNAbs) to promote viral neutralization and Fc-mediated clearance [123]. Illustration by Jill Gregory. Used with permission of ©Mount Sinai Health System.

Another strategy for overcoming Env antigenic heterogeneity is the use of small-molecule Env antagonists that reshape the conformational landscape of Env (Figure 4). By shifting the equilibrium between distinct Env states, these compounds alter antibody accessibility across multiple Env isoforms. CD4 mimetic compounds (CD4mcs) bind to the CD4-binding site and promote transitioning toward more open Env conformations (Figure 4B) [117,118,119]. Consistent with this mechanism, indoline- and piperidine-based CD4mcs have been shown to expose CD4-induced epitopes, thereby sensitizing infected cells to antibody-dependent cellular cytotoxicity (ADCC) mediated by non-neutralizing antibodies [120,121]. Notably, this strategy has also been shown to reduce the latent reservoir in a humanized mouse model [122]. These findings highlight how CD4mcs not only remodel Env conformations to promote immune recognition of infected cells but also have implications for cure strategies aimed at eliminating the persistent reservoir populations.

In contrast to CD4mcs, which exposes open conformations of Env, attachment inhibitor fostemsavir (FTR) or its biologically active form temsavir [124], shifts Env populations toward the closed prefusion State 1 conformation [125]. By stabilizing this state and preventing the downstream conformational changes required for viral entry, temsavir may reduce conformational heterogeneity by enriching the population of Env trimers occupying a common antigenic state (Figure 4B). The BRIGHTE study and follow-up trials demonstrated that FTR achieves durable viral suppression in heavily treatment-experienced individuals while also supporting immune recovery [126,127,128]. Temsavir can also reduce shedding of soluble gp120, which can trigger cytokine responses and promote bystander immune effects [129]. Additionally, FTR treatment of highly treatment-experienced individuals was recently shown by Benlarbi et al. to reduce the titer of antibodies that recognize CD4-induced (CD4i) epitopes [130]. Together, these findings further support the concept that small molecules can directly influence the conformational state and antigenic profile of Env displayed on infected cells in vivo, and their therapeutic effects.

Recent work also highlights that regions of gp41 critically shape how Env responds to these small molecules. Chatterjee et al. reported that a single residue in gp41′s heptad repeat region co-evolves with key gp120 residues and modulates Env trimer stability and susceptibility to small-molecule inhibitors, including temsavir and the CD4mc BNM-III-170 [131]. These findings support a model in which structural features of gp41 allosterically influence Env conformation and shape the susceptibility of Env to therapeutic intervention.

Together, these strategies suggest that Env modulators could be integrated with combination immunotherapy or vaccine-induced antibodies to reshape the antigenic landscape of Env and improve recognition of infected cells and circulating virions. Beyond blocking viral entry, these compounds actively reshape the antigenic landscape of Env by altering conformational state occupancy and the relative abundance of distinct Env populations. Consequently, by influencing conformational state occupancy and cleavage-dependent Env composition, FTR and related molecules may therefore serve not only as antiviral agents but also as adjunctive tools to potentiate antibody-based therapies.

## 6. Conclusions

Developing a cure for HIV will require both clearance of infected cells and prevention of new infection. As the sole viral protein displayed on infected cells and virions, Env remains the principal target of antibody-mediated responses. However, Env is a highly dynamic protein whose conformational landscape is shaped by its intrinsic structural flexibility and further refined by cleavage and glycan maturation. Increasing evidence further suggests that intracellular trafficking pathways and organization of the cytoplasmic tail contribute to the distribution of distinct Env populations that participate in cell-free and cell-to-cell viral transmission. In this framework, Env heterogeneity is not merely a byproduct of inefficient processing but a regulated feature that shapes antigenic exposure and immune recognition. These insights underscore the need to consider Env as a dynamic and spatially organized population, rather than a single structural entity, with important implications for the design of vaccines and therapeutic strategies aimed at effectively targeting infected cells and virions.

## Data Availability

No new data were created or analyzed in this study. Data sharing is not applicable to this article.

## Abbreviations

The following abbreviations are used in this manuscript:

HIV	Human immunodeficiency virus
Env	Envelope glycoprotein
bNAbs	Broadly neutralizing antibodies
CT	Cytoplasmic tail
ERC	Endosomal recycling complex
VS	Virological synapse
FIP1C	Family interacting protein 1C
TGN	Trans-Golgi network
NMR	Nuclear magnetic resonance
TMD	Transmembrane domain
LLPs	Lentiviral lytic peptides
KS	Kennedy sequence
MA	Matrix
smFRET	Single-molecule fluorescence resonance energy transfer
T/F	Transmitted/Founder
IP	Isoleucine-to-proline substitution
mAb	Monoclonal antibody
CD4mc	CD4 mimetic compounds
ADCC	Antibody-dependent cellular cytotoxicity
FTR	Fostemsavir
CD4i	CD4i induced
nNAb	Non-neutralizing antibody
